# Growth-Promoting Role of the miR-106a∼363 Cluster in Ewing Sarcoma

**DOI:** 10.1371/journal.pone.0063032

**Published:** 2013-04-26

**Authors:** Layne Dylla, Paul Jedlicka

**Affiliations:** 1 Cancer Biology Graduate Program, University of Colorado Denver, Anschutz Medical Campus, Aurora, Colorado, United States of America; 2 Medical Scientist Training Program, University of Colorado Denver, Anschutz Medical Campus, Aurora, Colorado, United States of America; 3 Department of Pathology, University of Colorado Denver, Anschutz Medical Campus, Aurora, Colorado, United States of America; Hemocentro de Ribeirão Preto, HC-FMRP-USP, Brazil

## Abstract

MicroRNAs (miRs) have been identified as potent regulators of both normal development and the hallmarks of cancer. Targeting of microRNAs has been shown to have preclinical promise, and select miR-based therapies are now in clinical trials. Ewing Sarcoma is a biologically aggressive pediatric cancer with little change in clinical outcomes despite improved chemotherapeutic regimens. There is a substantial need for new therapies to improve Ewing Sarcoma outcomes and to prevent chemotherapy-related secondary sequelae. Most Ewing Sarcoma tumors are driven by the EWS/Fli-1 fusion oncoprotein, acting as a gain-of-function transcription factor causing dysregulation of a variety of targets, including microRNAs. Our previous studies, and those of others, have identified upregulation of miRs belonging to the related miR-17∼92a, miR-106b∼25, and miR-106a∼363 clusters in Ewing Sarcoma. However, the functional consequences of this have not been characterized, nor has miR blockade been explored as an anti-cancer strategy in Ewing Sarcoma. To simulate a potential therapeutic approach, we examined the effects of blockade of these clusters, and their component miRs. Using colony formation as a read-out, we find that blockade of selected individual cluster component miRs, using specific inhibitors, has little or no effect. Combinatorial inhibition using miR “sponge” methodology, on the other hand, is inhibitory to colony formation, with blockade of whole clusters generally more effective than blockade of miR families. We show that a miR-blocking sponge directed against the poorly characterized miR-106a∼363 cluster is a particularly potent inhibitor of clonogenic growth in a subset of Ewing Sarcoma cell lines. We further identify upregulation of miR-15a as a downstream mechanism contributing to the miR-106a∼363 sponge growth-inhibitory effect. Taken together, our studies provide support for a pro-oncogenic role of the miR-106a∼363 cluster in Ewing Sarcoma, and identify miR-106a∼363 blockade, as well as miR-15a replacement, as possible strategies for inhibition of Ewing Sarcoma growth.

## Introduction

Ewing Sarcoma (EWS) is the second most common solid bone and soft tissue malignancy in children and young adults. With mesenchymal progenitor cells (MPCs) as the presumed cell of origin, the vast majority of Ewing Sarcoma tumors are driven by EWS/Ets fusion oncogenes, with the EWS/Fli-1 fusion, arising from the t(11,22)(q24:12) translocation, being the most common. [1], [2] EWS/Fli-1 is a non-physiologic, gain-of-function transcription factor that activates and represses a number of pathways important for tumorigenesis, including the Insulin Growth Factor-1 Receptor (IGF-1R) pathway, Cyclin D1 and p21 regulation of cell cycle progression, and TGFβ signaling. [3] More recently, a number of studies, including ours, have identified alterations in microRNA expression in Ewing Sarcoma. [4]–[8] Unlike other pediatric tumors, the five-year survival rates in Ewing Sarcoma have remained relatively unchanged over the last thirty years. [9] While EWS is a chemosensitive-tumor, these treatments place survivors at high risk for long-term sequelae such as poor growth, infertility, endocrine dysfunction, and secondary malignancies. [10] Thus, innovative, targeted therapies are needed to improve primary outcomes and long-term quality of life. [11] However, with the exception of IGF-1R targeted blockade, understanding EWS/Fli-1-mediated tumorigenesis has yielded few therapeutic alternatives to date. [12], [13] Furthermore, despite the promise of IGF-1R targeted therapy, many EWS patients either fail to respond to treatment or develop resistance. One alternative avenue of targeted therapies with pre-clinical promise in a number of cancers, but relatively unexplored in Ewing Sarcoma, is modulation of microRNAs [14]–[16].

MicroRNAs (miRs) are short non-coding RNAs that bind a 2–7 nucleotide sequence (“seed sequence”) within the 3′ untranslated regions (UTR) to mediate gene repression via degradation or sequestration of the targeted mRNA. [17] MiRs have tremendous regulatory power, potentially regulating over 60% of the genome, including regulation of normal cellular functions and the hallmarks of cancer. [18] The exact role of any given miR depends on the cell type and possibly other, currently largely unknown, regulatory factors. Several studies examining miR alterations in Ewing Sarcoma have identified upregulation of miRs belonging to three paralogous clusters – miR-17∼92a, miR-106b∼25, and miR-106a∼363. [4]–[6] These clusters are highly conserved across species, are thought to have arisen from genetic duplications during evolution, and have been shown to be pro-oncogenic in a number of malignancies. [19]–[27] In EWS, tumor expression of multiple members of these clusters has recently been show to be negatively correlated with both 5-year event-free survival and overall survival. [28] Thus, manipulation of these clusters, or their component miRs, may have therapeutic benefit in Ewing Sarcoma. The purpose of the present study was to determine the requirements for miR cluster upregulation in Ewing Sarcoma, and identify strategies for their blockade to inhibit Ewing Sarcoma oncogenesis.

## Results

### The miR-17∼92a, miR-106b∼25, and miR-106a∼363 Clusters are Overexpressed in Ewing Sarcoma Cell Lines Compared to Human Mesenchymal Progenitor Cells, the Presumed Cells of Ewing Sarcoma Origin

**Figure 1:**
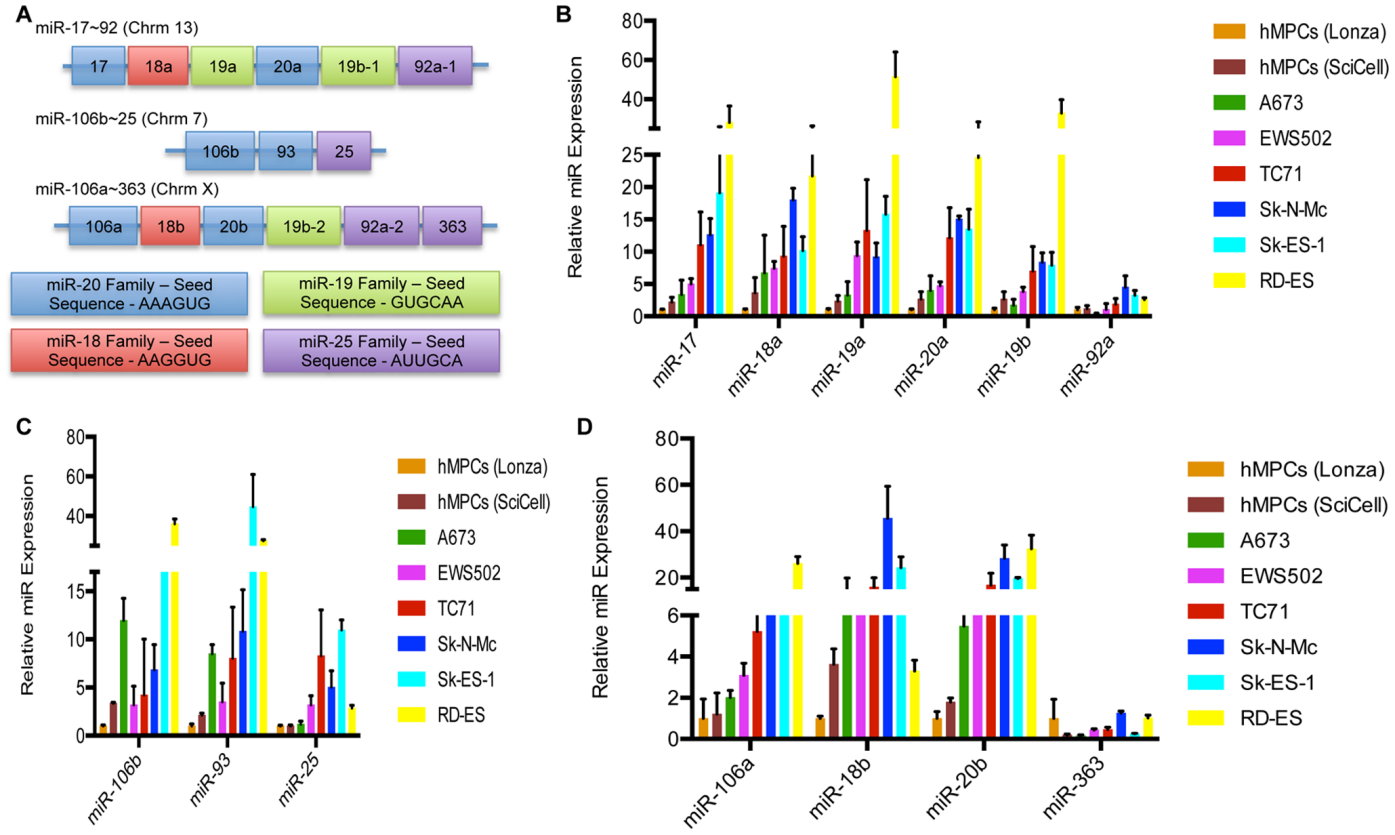
The miR-17∼92, miR-106b∼25, and miR-106a∼363 clusters are overexpressed in EWS cell lines compared to hMPCs. (A) The miR-17∼92a, miR-106b∼25, and miR-106a∼363 paralogous clusters and their respective miR families. (B–D) Expression levels of mature miRs belonging to the miR-17∼92a (B), miR-106b∼25 (C), and miR-106a∼363 (D) clusters in hMPCs and EWS cell lines was determined by qRT-PCR. Values represent the mean and standard deviation of biological triplicates.

**Figure 2:**
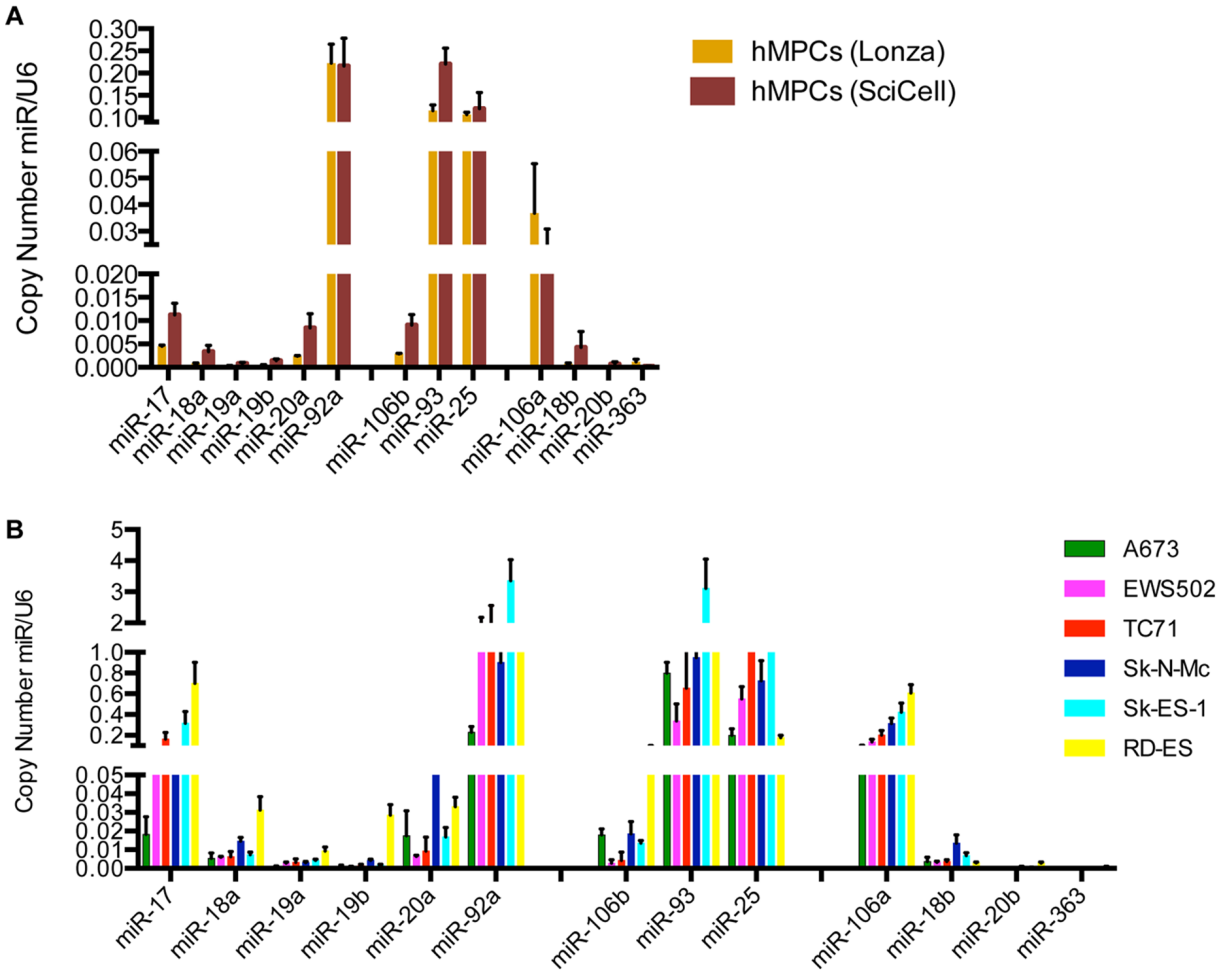
Relative levels of miR-17∼92a, miR-106b∼25, and miR-106a∼363 cluster components in EWS cells and hMPCs. Relative levels of the individual miRs within a given cluster in each cell line were determined by qRT-PCR using a best-fit linear equation generated by the amplicon standard curve in order to account for differences in primer efficiency. [57] Values represent the mean and standard deviation of biological triplicates.

Our previous studies profiling miRs downstream of the EWS/Fli1 fusion oncoprotein identified several members of the miR 17∼92a, 106b∼25 and 106a∼363 clusters as candidate upregulated miRs in Ewing Sarcoma. [4] These miR clusters have been demonstrated to play important roles, largely pro-oncogenic, in a broad array of cancers. [19]–[27] We were thus very interested in further understanding their potential role in Ewing Sarcoma. We began by surveying expression levels of all the component miRs in a panel of Ewing Sarcoma cell lines (A673, EWS502, TC71, Sk-N-Mc, Sk-ES-1, and RD-ES) relative to two different human mesenchymal progenitor cell (hMPC) lines, the presumed cells of Ewing Sarcoma origin, using qRT-PCR (Figure 1b–d). Overall, we found that Ewing Sarcoma cell lines consistently upregulate all the cluster component miRs, with the exception of miR-363, which was only minimally expressed in both EWS cells and hMPCs. Some cell line differences were evident, with A673 and EWS502 cells tending toward lower levels of upregulation, and TC71, Sk-N-Mc, Sk-ES-1 and RD-ES cells tending toward greater upregulation of the clusters. We next compared the relative expression levels of individual miR cluster members within the various cell lines, by correcting for any differences in qRT-PCR primer efficiency and examining the absolute copy number of each miR relative to the copy number of an endogenous U6 RNA control. This revealed miR-92a, miR-93, miR-25, and miR-106a to be the most highly expressed miRs between studied miRs in both hMPCs and EWS cell lines (Figure 2a and 2b).

### MiR Blocking Experiments Identify a Growth-promoting Role for the 106a∼363 Cluster in Ewing Sarcoma

We next sought to determine the functional consequences of miR overexpression in Ewing Sarcoma. Overexpression of the miR clusters and/or component miRs has been shown to promote growth in a number of cancer types. [22], [25]–[27], [29]–[35] We thus postulated that miR cluster overexpression would be growth-promoting in Ewing Sarcoma. We began by examining the effects of blockade of select individual cluster component miRs. We chose a clonogenic assay as a stringent assay of cell growth to screen for effects of miR inhibition. MiR inhibition was achieved using either hairpin inhibitor (HI) or locked nucleic acid (LNA) methodology, and verified using the psiCHECK2 dual luciferase reporter system. We compared these two methodologies (HI and LNA) for select miRs and did not observe differences in the effectiveness of miR blockade (Figure S1). Inhibition of miRs 17, 92a, 93 and 25 was chosen because of the robust overexpression of these miRs in EWS cells compared to hMPCs and/or their high expression levels in both cell types. In addition, miR-19b inhibition was also selected because, while not expressed at high levels, this miR has been shown in other systems to be sufficient for the pro-oncogenic activity of the entire miR-17∼92a cluster. [25], [29], [36] Sk-ES-1 cells were chosen because of their tendency toward high miR cluster overexpression. Individual blockade of miR-93, miR-25, miR-17, miR-19b, or miR-92a did not show a significant effect on Sk-ES-1 clonogenic growth (Figure S2). Individual blockade of miRs 25 and 93 was also tested in two other EWS cell lines (TC71 and Sk-N-MC), but, similar to the findings in Sk-ES-1 cells, did not significantly affect clonogenic growth (Figure S2b–c). Inhibition of miRs 25 and 93 in combination tended to show a very slight inhibition of growth in some experiments, but this did not reach statistical significance (Figure S2a). In all cases, the dosage of miR inhibitor used appeared to be sufficient to effect miR blockade in Sk-ES-1 cells, as determined using the psiCHECK2 reporter system (Figure S2d and S2f).

**Figure 3:**
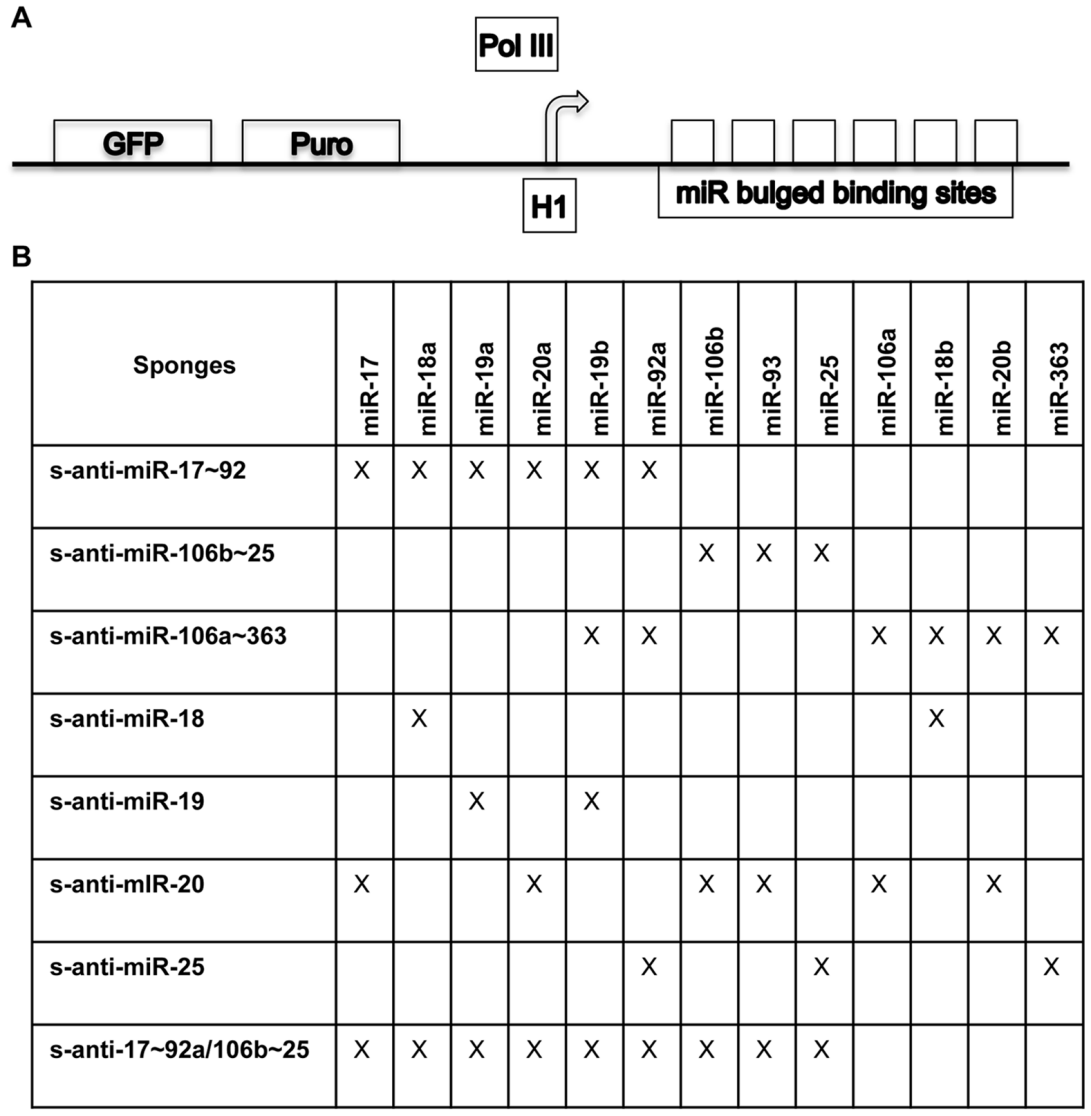
Design of miR-blocking sponges. (A) pGreen lentiviral vector containing the HI PolII promoter driving the expression of multiple complementary bulged microRNA binding sites. Each binding site is complementary to a single microRNA with the exception of four mismatchs that create a bulge to promote sponge stability. [37] Separate promoters drive GFP and puromycin selection markers. (B) Table listing the individual miRs targeted by the various microRNA sponge constructs.

**Figure 4:**
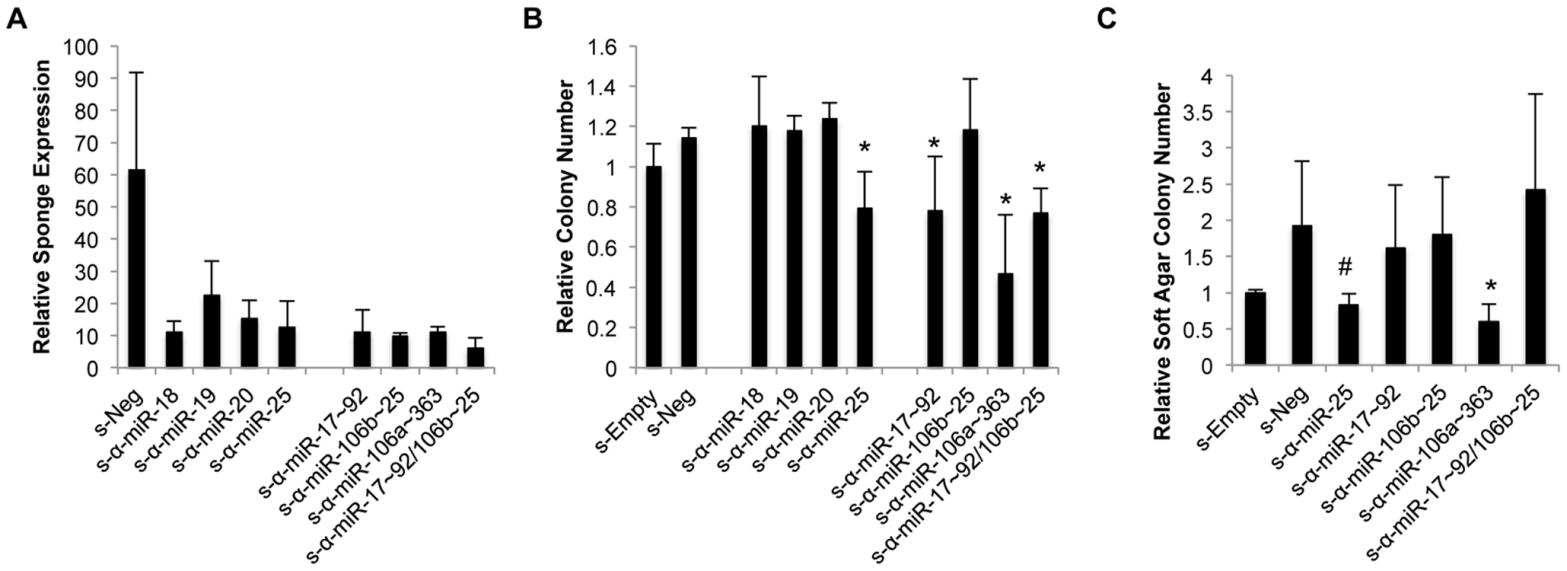
Effects of miR-blocking sponge expression on Ewing Sarcoma cell growth. (A) Sponge expression levels in Sk-ES-1 cells as determined by qRT-PCR. Results represent the mean and SEM of three independent experiments, each performed in triplicate. (B) Colony formation by Sk-ES-1 cells stably transduced with the indicated miR-blocking sponge constructs, in a clonogenic assay. Data represent the mean and SEM of two independent experiments, each performed in triplicate. *p<0.05 compared to s-Empty and s-Neg, individually. (C) Colony formation by Sk-ES-1 cells stably transduced with the indicated miR-blocking sponge constructs, in a soft agar assay. Results represent the mean and SEM of three independent experiments, each performed in triplicate. *p<0.05 compared to s-Empty and s-Neg, individually; ^#^p<0.05 compared to s-Empty only based on an unpaired student’s t-test.

Given the lack of effects by targeting individual miRs, but the possible slight effect seen by inhibition of multiple miRs, we examined the possibility that miRs may be acting cooperatively. MicroRNAs can be grouped into clusters containing miRs that are co-expressed based on their locations within the genome, as well as into families, which contain miRs with a shared seed sequence. We explored the possibility that miRs with related seed sequences from different clusters compensate for each other, or, alternatively, that miRs unrelated by sequence, but co-expressed from the same cluster, may work together. To further probe this in Ewing Sarcoma, and distinguish between these two scenarios, we took advantage of the recently developed miR “sponge” technology, which allows simultaneous blockade of multiple miRs. [37] Specifically, an RNA molecule is engineered with miR complementary sequence resembling a perfect target, including a mismatched bulge to prevent degradation of the miR-RNA duplex. This system has been used effectively in a number of studies to understand miR function. [37] For our experiments, we employed the lentiviral pGreen expression system to drive sponge expression; this system uses the RNA polymerase III-driven H1 promoter driving the expression of multiple bulged miR binding sites. The modular composition of the different miR sponge constructs used is shown in Figure 3. Specifically, the sponge constructs generated were: s-α-miR-18, s-α-miR-19, s-α-miR-20, and s-α-miR-25, to target all the members of the specified miR family, as defined by shared seed sequence; and s-α-miR-17∼92a, s-α-miR-106b∼25, and s-α-miR-106a∼363, to target all the members of a given miR cluster. Additionally, given the established importance of the miR-17∼92a and miR-106b∼25 clusters in other cancers, the combination sponge construct s-α-17∼92a/106b∼25 was generated to target these clusters in tandem. For negative controls, two different constructs were used, s-Empty, lacking any miR-binding sites, and s-Neg, a non-targeting control that is based on a sequence that no miRs are predicted to bind, but that still resembles a potential miR binding site, as done by others previously. [37] Sk-ES-1 cells were again selected for screening experiments, as a cell line with robust endogenous miR cluster overexpression (see Figure 1). Cells were stably infected with the above constructs and, following antibiotic (Puromycin) selection, levels of expression of each were determined by qRT-PCR, using the same primer pair to permit comparison between constructs (Figure 4a). These analyses revealed all of the miR-targeting sponges to be expressed at comparable levels to each other. The relative level of the s-Neg control was more variable and overall higher than the levels of the sponge constructs, possibly reflecting some degree of negative selection against the miR-targeting constructs.

We next examined the phenotypic effects of miR-blocking sponge expression on Sk-ES-1 clonogenic cell growth (Figure 4b). Clonogenic growth was similar for both the s-Empty and s-Neg expressing cells. Of the sponges targeting miR families (with related seed sequence), only s-α-miR-25 had an effect on clonogenic growth, causing ∼20% decrease in colony formation, compared to both controls (s-Empty and s-Neg). This was interesting given the absence of a phenotype upon specific blockade of miRs 25 and 92a alone (see Figure S2), suggesting that members of the miR-25 family may indeed be able to compensate for one another. The other miR family targeting sponges, s-α-miR-18, s-α-miR-19 and s-α-miR-20, had no significant effect on clonogenic growth of Sk-ES-1 cells. Of the miR cluster targeting sponges (targeting co-expressed miRs, but with unrelated seed sequences), the one targeting the miR-17∼92a cluster caused ∼20% decrease in colony formation. The sponge targeting the miR-106b∼25 cluster had no effect on clonogenic growth. Furthermore, the effect on growth of the combination miR-17∼92a/miR-106b∼25 sponge was essentially identical to the miR-17∼92a targeting sponge. Interestingly, of the cluster-targeting sponges, the one targeting the miR-106a∼363 cluster showed the most potent inhibitory effect, with an average 50% decrease in clonogenic growth. The s-α-miR-106a∼363 sponge also yielded the most potent inhibition of colony formation in a soft agar assay of anchorage-independent growth (Figure 4c). To verify that the s-α-miR-106a∼363 sponge was acting through a miR-blocking mechanism, a construct was generated containing mutated seed sequence for each of the miRs in the cluster. Stable expression of this seed-mutated sponge construct resulted in loss of inhibition of clonogenic growth relative to the (non-mutated) s-α-miR-106a∼363 sponge in Sk-ES-1 cells (Figure S3). Thus, miR cluster blockade appears to be overall more effective as a means of inhibiting cell growth than miR family blockade in Ewing Sarcoma, with blockade of the miR-106a∼363 cluster showing the highest potency.

**Figure 5:**
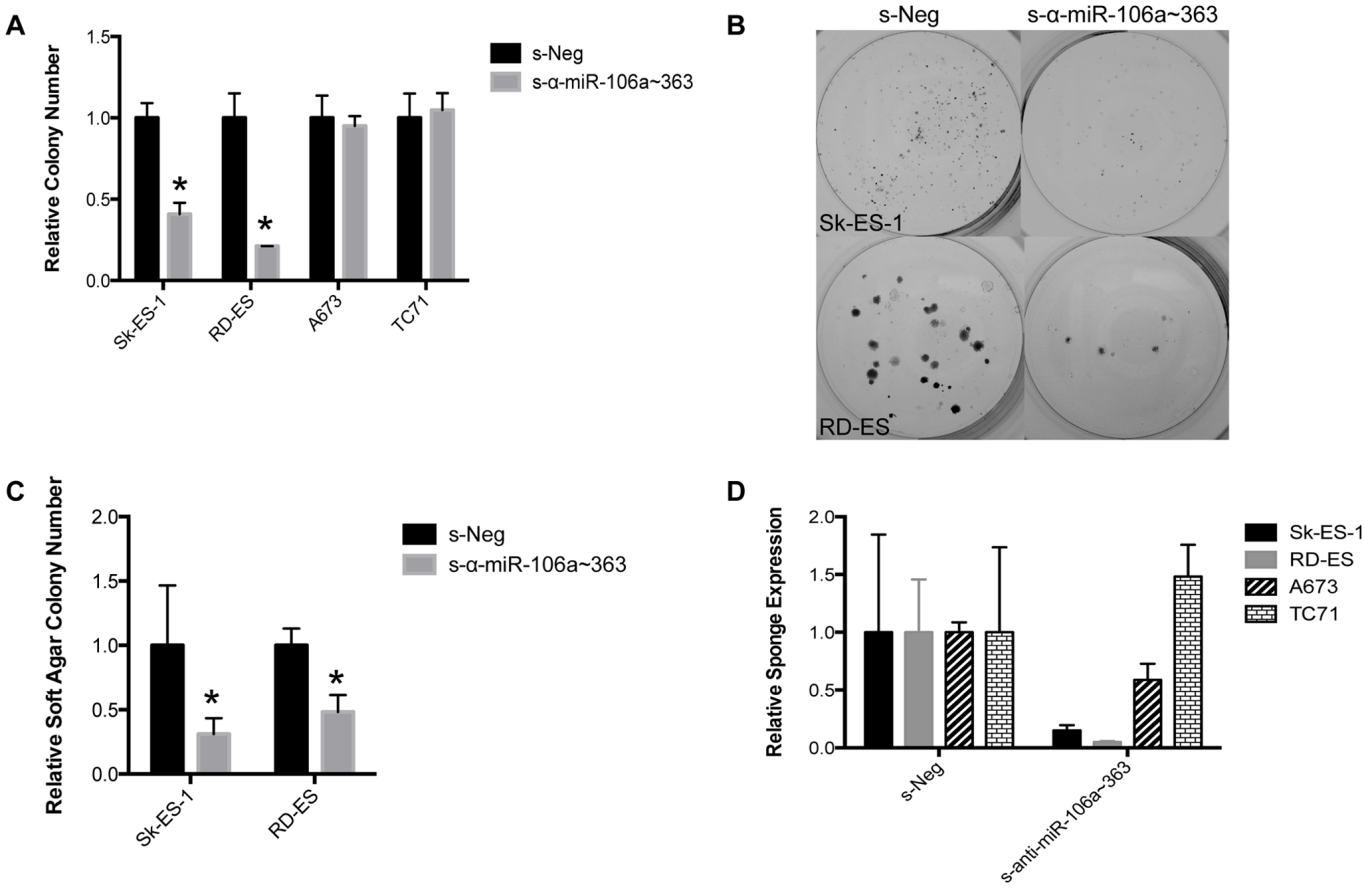
Growth effects of the s-α-miR-106a∼363 sponge in other Ewing Sarcoma cell lines. Comparison of colony formation in a panel of EWS cell lines (Sk-ES-1, RD-ES, A673, and TC71) stably transduced with s-Neg or s-α-mir-106a∼363, in a clonogenic assay (A) and in a soft agar assay (B). Results represent the mean and SEM of a minimum of three independent experiments, each performed in triplicate. *p<0.05 compared to s-Neg based on an unpaired student’s t-test. (C) Sponge expression in EWS cell lines was determined by qRT-PCR. Data represent the mean and SEM of a minimum of three independent experiments, each performed in triplicate.

We next tested the inhibitory activity of the s-α-miR-106a∼363 sponge in additional EWS cell lines. Similar to Sk-ES-1 cells, stable expression of s-α-miR-106a∼363 inhibited both clonogenic and anchorage-independent growth of RD-ES cells (Figure 5a–c). Introduction of the s-α-miR-106a∼363 sponge into A673 or TC71 cells, on the other hand, did not affect clonogenic growth. Interestingly, both cell lines vulnerable to the sponge effect appeared to restrict sponge expression (Sk-ES-1 and RD-ES), further suggesting that introduction of s-α-miR-106a∼363 is deleterious in these cells (Figure 5d).

### Upregulation of miR-15a Contributes to the Growth Inhibitory Effects of miR-106a∼363 Blockade in Ewing Sarcoma

**Figure 6:**
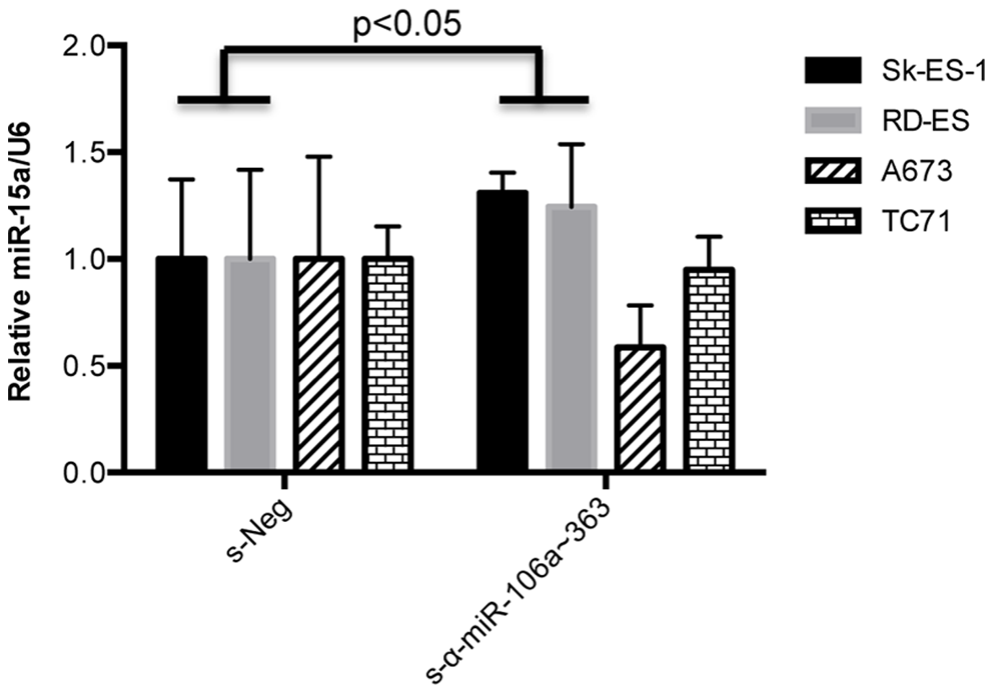
Effects of s-α-miR-106a∼363 expression on miR-15a levels in Ewing Sarcoma cell lines. Levels of miR-15a in the indicated Ewing Sarcoma cell lines stably expressing s-Neg or s-α-miR-106a∼363, as determined by qRT-PCR. Results represent the mean and SEM of a minimum of four independent experiments, each performed in triplicate.

MicroRNAs have many targets in the cell, and the relative importance of individual miR-target interactions varies among cell types. A number of targets have been identified for the miR-17∼92a and miR-106b∼25 clusters in other cancers. Less is known about the biology of the miR-106a∼363 cluster and few miRs have been functionally evaluated in sarcomas. To probe for relevant mechanisms of action of the s-α-miR-106a∼363 sponge in Ewing Sarcoma, we examined the expression of a number of targets, and/or related pathway activity, of miR-106a∼363, as well as the closely related miR-17∼92a, cluster, which have been identified in other cancers as regulators of growth and apoptosis. These targets/pathways included PTEN and the PI3K/Akt pathway, Erk1/2 and MAPK signaling, TGFβ signaling, and Wnt signaling. However, this approach failed to reveal a candidate mechanism of s-α-miR-106a∼363 sponge action. We thus employed an unbiased approach to identify targets and/or pathways affected by s-α-miR-106a∼363 sponge expression in Ewing Sarcoma. Expression profiling using Affymetrix whole transcript arrays was performed on Sk-ES-1 cells expressing either s-Neg or s-α-miR-106a∼363. Interestingly, this analysis revealed the transcript containing miR-15a as significantly upregulated in the context of s-α-miR-106a∼363 expression (Figure S4). MiR-15a has been shown to be growth inhibitory in a number of cancers. [38]–[42] Subsequent qRT-PCR analysis showed miR-15a to be increased upon s-α-miR-106a∼363 expression in responsive cell lines (SK-ES-1 and RD-ES), but not in the unresponsive cell lines (TC71 and A673) (Figure 6). We thus pursued miR-15a upregulation as a possible mechanism contributing to the s-α-miR-106a∼363 growth-inhibition phenotype in Ewing Sarcoma cells.

**Figure 7:**
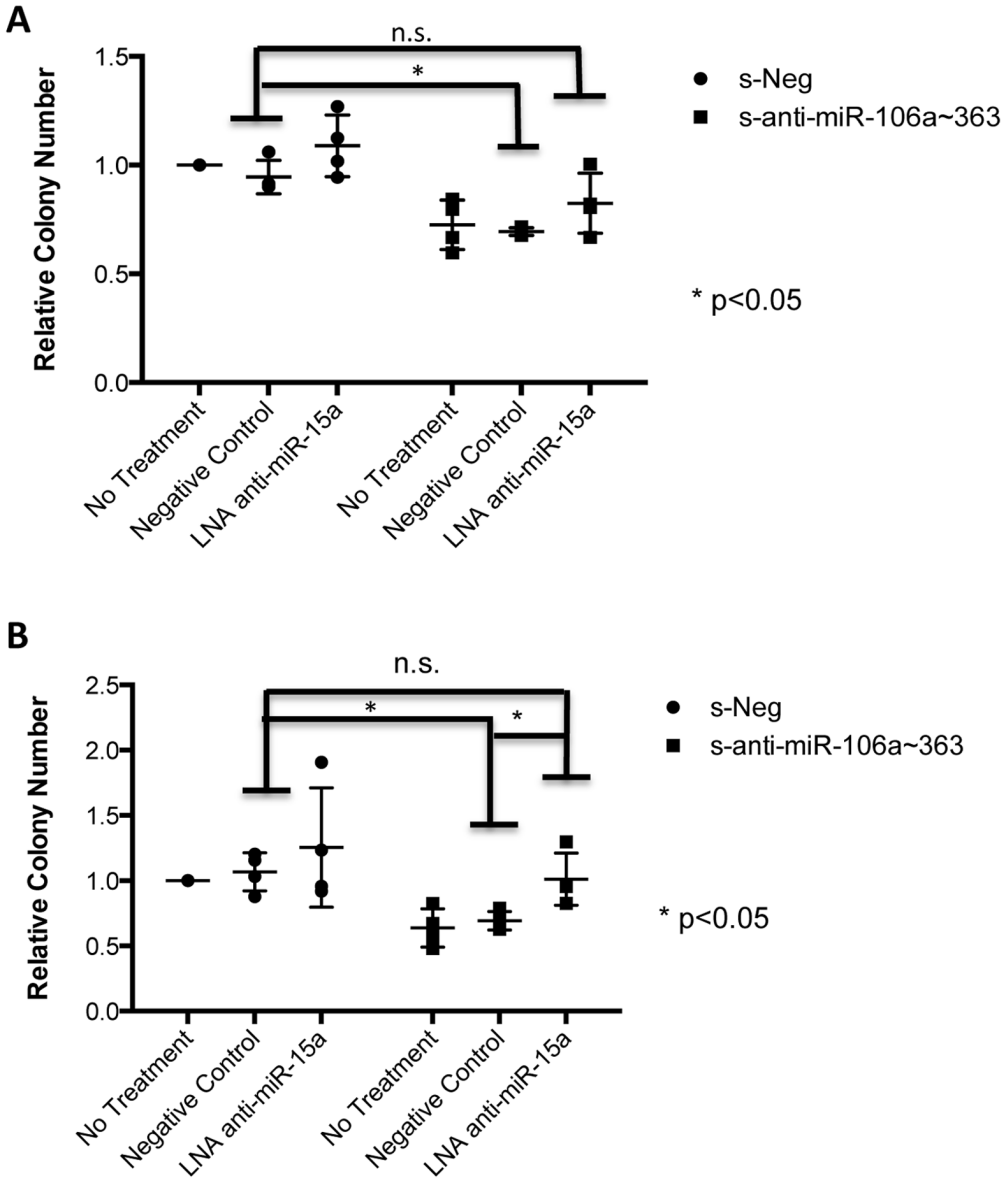
MiR-15a blockade reverses growth inhibition by s-α-miR-106a∼363. Clonogenic growth assay in Sk-ES-1 (A) and RD-ES (B) cells stably expressing s-Neg or s-α-miR-106a∼363 that were untreated or treated with 50 nM of a negative control LNA or an LNA targeting miR-15a. Results represent the mean and SEM of three independent experiments, each performed in triplicate. *p<0.05; n.s. = not significant; based on an unpaired student’s t-test.

**Figure 8:**
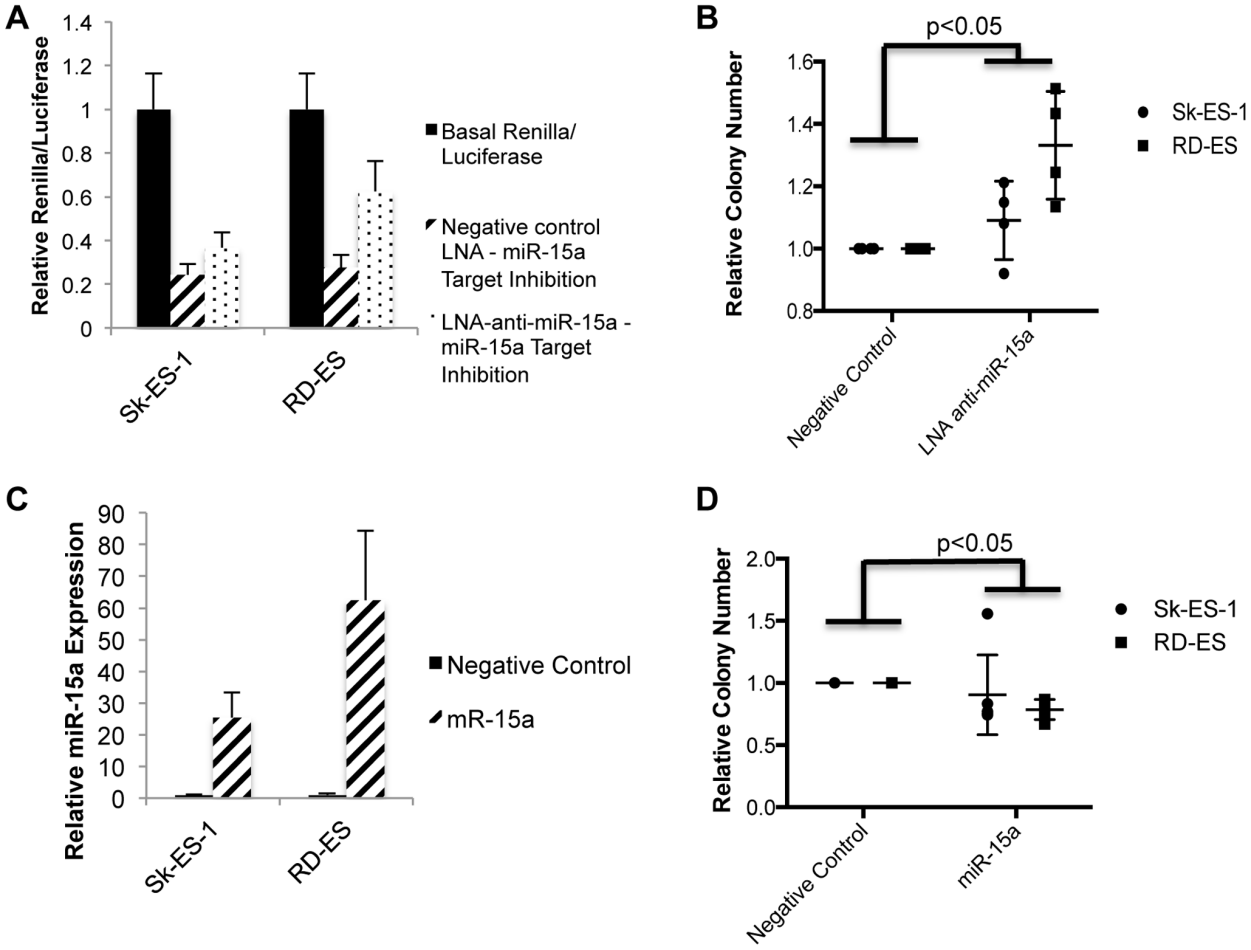
MiR-15a inhibits EWS clonogenic growth. (A) Dual Renilla/Luciferase assay performed in Sk-ES-1 and RD-ES cells transfected with 50 nM LNA-anti-miR-15a or a negative control LNA, and the psiCHECK2 dual luciferase reporter with a miR-15a complementary binding site, or non-targeting binding site, in the 3′ UTR. Results represent the mean and SEM of two independent experiments, each performed in triplicate. “Basal” reporter activity corresponds to cells transfected with non-targeting reporter and negative control (non-targeting) LNA. (B) Clonogenic assay in Sk-ES-1 and RD-ES cell lines treated with 50 nM of a negative control LNA or an LNA targeting miR-15a. (C) Relative overexpression of miR-15a in Sk-ES-1 and RD-ES cells treated with 25 nM of a negative control mimic or miR-15a mimic was determined by qRT-PCR. miR-15a levels were normalized to an endogenous U6 control. Results represent the mean and SEM of three independent experiments, each performed in triplicate. (D) Clonogenic assay in Sk-ES-1 and RD-ES cell lines treated with 25 nM of a negative control mimic or a miR-15a mimic. Statistical significance was determined based on an unpaired student’s t-test comparison between the indicated treatments.

We used an LNA approach to determine whether miR-15a blockade can reverse the growth-inhibitory effect of the s-α-miR-106a∼363 sponge. In both Sk-ES-1 and RD-ES cells, treatment with LNA-anti-miR-15a resulted in at least partial rescue of growth inhibition by s-α-miR-106a∼363 (Figure 7). The effect was more robust in RD-ES cells where colony formation by s-α-miR-106a∼363/anti-miR-15a LNA cells was both similar to s-Neg/negative control LNA cells, and statistically greater than colony formation by s-α-miR-106a∼363/negative control LNA cells (Figure 7b). In Sk-ES-1 cells, colony formation by s-α-miR-106a∼363/anti-miR-15a LNA cells was similar to colony formation by s-Neg/negative control LNA cells; however, while, there was a trend toward increased colony formation relative to the s-α-miR-106a∼363/negative control LNA group, this did not reach statistical significance (Figure 7a). Interestingly, LNA inhibition of miR-15a, as determined using the psiCHECK2 reporter system, was more potent in RD-ES cells (Figure 8a), suggesting this as the possible basis for the more robust rescue in this cell line. Taken together, these findings suggest that miR-15a upregulation contributes to the growth-inhibitory effects of miR-106a∼363 blockade in Ewing Sarcoma.

To further explore the role of miR-15a in Ewing Sarcoma, we asked how miR-15a manipulation affects clonogenic growth in the absence of miR-106a∼363 inhibition. Inhibition of miR-15a in Sk-ES-1 and RD-ES cells, using the same LNAs as above, resulted in increased clonogenic growth compared to a non-targeting negative control LNA (Figure 8b). As in the rescue experiments above, LNA-anti-miR-15a treatment resulted in a greater increase in colony formation in RD-ES cells compared to Sk-ES-1 cells. Again, this may be due in part to greater potency of LNA-mediated miR-15a inhibition in RD-ES cells compared to Sk-ES-1 cells (Figure 8a). Conversely, transient overexpression of miR-15a, using a miR mimic, resulted in reduction of Sk-ES-1 and RD-ES clonogenic growth compared to a scrambled negative control miR mimic (Figure 8c and 8d). As in the LNA experiments, the more potent phenotypic effect of miR-15a mimic treatment in RD-ES cells correlated with higher miR-15a overexpression levels in this cell line. These findings further support a growth suppressive role of miR-15a in Ewing Sarcoma.

## Discussion

Targeting of miRs through delivery of LNAs or antagomiRs has recently been identified to have substantial promise in pre-clinical models, due to the specificity and relative lack of off-target effects as are seen with traditional chemotherapeutics. Currently, one LNA-based therapy is in phase 2 clinical trials and showing substantial promise. [43] Our previous studies identified members of the three paralagous oncomiR clusters, miR-17∼92a, miR-106b∼25, and miR-106a∼363, among the most strongly EWS/Fli1-upregulated miRs. [4] Other miR profiling studies in Ewing Sarcoma have made similar observations, and also verified oncomiR cluster overexpression in patient tumors. [5]–[8] Moreover, in a recent study, overexpression of several members of these clusters has been shown to correlate with both poor 5-year event free survival and overall survival. [28] Similarly, in Alveolar Rhabdomysarcoma, another pediatric sarcoma, tumor expression of multiple members of the theses clusters has been shown to be negatively correlated with patient prognosis. [24] In the present study, we undertook the first functional analysis of these clusters in Ewing Sarcoma, as well as the first systematic analysis of the effects of blockade of miR genomic clusters versus their seed sequence-related components. These studies have identified an important role for the miR-106a∼363 cluster in the promotion of clonogenic and anchorage-independent growth in Ewing Sarcoma, and have uncovered miR-15a as a novel contributor to oncomiR cluster action in cancer.

MicroRNAs can be grouped into miR clusters based on their locations within the genome or into miR families based on the presence of shared seed sequences. Within a given family, most miRs differ by only a few nucleotides, leaving the potential for redundancy among family members. There is also a high degree of similarity between the miR-17∼92a and miR-106a∼363 clusters, with both miR-19b and miR-92a being absolutely duplicated between the two clusters. To date, there have been few systematic analyses of these clusters and their component miR families. In developmental studies, the miR-106a∼363 cluster is dispensable for normal mouse development. [44] However, double knock out experiments with just the miR-17∼92a and the miR-106a∼363 cluster were not performed, only the triple knock out experiment for all three clusters and the double knock out for both miR-17∼92a and miR-106b∼26. This leaves the possibility that the exaggerated developmental phenotypes seen in the miR-17∼92a/miR-106b∼25/miR-106a∼363 triple knock out mice could still be partially contributed to by the loss of the miR-106a∼363 cluster. Our data using microRNA-blocking sponges to examine the effects of inhibition of entire miR clusters compared to inhibition of miR families, the first analysis of this type in a pediatric cancer, suggest that, at least in Ewing Sarcoma, inhibition of all the members of a given cluster may be more potent than inhibition of paralogous miRs with shared seed sequences. This in turn suggests that the co-expressed functionally distinct miRs cooperates to promote oncogenesis and that targeting these miRs together may provide the most potent blockade of oncogenesis.

The miR-17∼92a cluster has been implicated in numerous cancer types, while the role of the miR-106a∼363 in cancer is at present relatively obscure. We were, thus, somewhat surprised by the more potent inhibitory activity of the miR-106a∼363-targeting sponge, relative to the sponge targeting the miR-17∼92a cluster. One possibility is that miR-106a∼363 is indeed more important than miR-17∼92a in Ewing Sarcoma pathogenesis. However, it is also possible that s-α-miR-106a∼363 and s-α-miR-17-92a cross-react, at least to some extent, given how closely related the miRs are from the two clusters. Thus, we cannot exclude the possibility that the s-α-miR-106a∼363 sponge works at least in part by blocking miRs from the miR-17∼92a cluster. Even if the sponges do to some extent cross-react, however, the more potent activity of s-α-miR-106a∼363 supports a role for the miR-106a∼363 cluster in Ewing Sarcoma.

While the miR-106a∼363 cluster is by far the least studied of the paralogs, an increasing number of studies are emerging which support the idea that it plays an important role in tumorigenesis. In a bronchial epithelial chemical carcinogenesis model and in gastric cancer models, miR-106a promotes both *in vitro* and *in vivo* tumorigenesis. [22], [31] Furthermore, three members of the miR-17 family (miR-17, miR-106b and miR-106a) each directly inhibit p21 in Diffuse Large B-cell Lymphoma and in Burkitt’s Lymphoma to increase cellular proliferation. [23] Importantly, in this context, specific blockade of miR-106a alone also resulted in increased expression of Bim and decreased expression of CDK4/CDK6. Together, these studies support an important and distinct role for miR-106a in tumorigenesis. Interestingly, in our studies, inhibition of miR-106a alone, or in combination with inhibition of miR-92a or miR-92a and miR-20b, using LNAs, did not significantly inhibit Sk-ES-1 clonogenic growth. This suggests that inhibition of the entire miR-106a∼363 cluster may be required for the growth-inhibitory effects of the s-α-miR-106a∼363 sponge in Ewing Sarcoma.

The growth inhibitory effects of s-α-miR-106a∼363 demonstrate cell specificity, affecting growth of Sk-ES-1 and RD-ES, but not A673 and TC71 cells. The degree of miR-106a∼363 overexpression (high in Sk-ES-1 and RD-ES cells, but lower in A673 cells) may explain the difference in phenotypes between these cell lines. However, other factors must be responsible for the lack of phenotype in TC71 cells, which manifest miR-106a∼363 overexpression resembling Sk-ES-1 and RD-ES cell. Interestingly, both Sk-ES-1 and RD-ES cells contain the EWS/Fli-1 type 2 fusion, while A673 and TC71 cells bear the EWS/Fli-1 type 1 fusion. This suggests the intriguing possibility that fusion type may be a contributory factor to the observed cell type specificity. The EWS/Fli-1 type 1 fusion accounts for approximately 60% of all EWS/Ets translocations, with the type 2 fusion accounting for an additional 25%. [45] These translocations differ in the number of exons from the C-terminus of Fli-1 (type 1 containing exons 6–9 and type 2 containing exons 5–9). Originally, EWS/Fli-1 fusion subtype was studied in part because a type 1 fusion appeared to confer a better prognosis. [46] Additionally, in mouse xenograft experiments, cell lines bearing the type 1 fusion had delayed tumor initiation and resulted in fewer primary tumors and metastases. [45] In microarray expression profiling of type 1 fusion compared to non-type 1 fusion bearing cells revealed 41 genes were differentially expressed, with all being downregulated in the non-type 1 fusion bearing cell lines. [47] These genes included genes involved in muscle development, proliferation, and calcium-ion binding, among others. However, the functional consequences of these differences have not been studied in detail. Moreover, more recent patient data suggest no difference in prognosis or chemosensitivity between type 1 and non-type 1 fusion-bearing tumors with current treatment protocols. [48] Our data suggest possible differences in microRNA biology between these groups, but this possibility awaits further experimental exploration.

Our studies demonstrate a growth-inhibitory role for miR-15a in Ewing Sarcoma. MiR-15a has been shown to be tumor suppressive in other systems, primarily through regulation of a variety of cell cycle targets, including Wee1 and multiple cyclins. [38], [39], [41], [42] In mouse genetic models, miR-15a deletion significantly accelerates the development of Chronic Lymphocytic Leukemia, in part through de-repression of multiple miR-15a-targeted cyclins (Cyclin D1 and D3, Cyclin E), CDK6 and the anti-apoptotic factor Bcl-2. [40], [49] In prostate cancer, inhibition of miR-15a leads to increased anchorage-independent growth and migration *in vitro*, as well as transforming a non-tumorigenic prostate cell line *in vivo*. [50] On the other hand, intratumoral injection of a miR-15a inhibitor induces tumor necrosis and regression. Again, the identified miR-15a targets included Cyclin D1 and Bcl-2. Not only does miR-15a negatively regulate cell cycle progression through inhibition of CDKs and cyclins, but its inhibition of two cyclin kinases, Wee1 and Chk1, is associated with Cisplatin resistance in cancer cell lines and this resistance is reversed upon re-expression of miR-15a. [42] Finally, in Ewing Sarcoma, treatment with MLN4924, a compound that inhibits neddylation, and subsequent degradation, of cullins in cullin-RING ubiquitin ligase complexes leading degradation of a variety of proteins, leads to a G2 cell cycle arrest and apoptosis. [51] Interestingly, two miR-15a targets, Wee1 and Cyclin E, were increased under such conditions, with Wee1 expression being required to mediate the G2 arrest. The exact mechanism of Wee1 accumulation was not determined. In our microarray expression profiling of s-Neg and s-α-miR-106a∼363 expressing Sk-ES-1 cells, GSEA phenotypic analysis revealed the KEGG cell cycle pathway as being differentially expressed. Somewhat unexpectedly, many genes in this pathway were overall upregulated by s-α-miR-106a∼363 expression, including two known miR-15a targets, Cyclin E1 and E2, and E2F1. This is not entirely surprising, however, in light of data from osteosarcoma, where E2F1, Cyclin E, and miR-15a form a complex regulatory loop, whereby E2Fs induce both Cyclin E and miR-15a expression, and miR-15a limits proliferation by inhibiting Cyclin E expression during G1/S. [41] The miR-17∼92 paralogous clusters, are also induced by E2F1 expression and can act to limit proliferation. [35], [52], [53] Thus, perturbation of individual components of these regulatory loops may have complex effects on the network as a whole.

Our studies indicate that miR-15a levels are also sensitive to perturbation of oncomiR cluster levels. The precise mechanism(s) of miR-15a upregulation by miR-106a∼363 blockade remain to be determined. MiR-15a can be regulated transcriptionally. [41], [54] However, we did not observe consistent increases in Dleu2, the miR-15a primary transcript, by qRT-PCR in s-α-miR-106a∼363 sponge expressing cells, arguing against a major role for a transcriptional mechanism. Interestingly, miR-15a expression in the mouse has recently been shown to be regulated by another microRNA, miR-709, at the level of processing, and similar mechanisms may play a role in Ewing Sarcoma. [55], [56].

In summary, we demonstrate that members of the miR-17∼92a, miR-106b∼25, and miR-106a∼363 clusters are upregulated in EWS. Our systematic functional analysis of these paralogous clusters, using miR blocking sponge methodology, identifies the miR-106a∼363 cluster as a potentiator of EWS growth. This potentiation manifests cell type specificity, possibly in part related to cellular context related to EWS/Fli1 fusion type, as well as likely other factors. In cell lines sensitive to the growth inhibitory effects of miR-106a∼363 blockade, modulation of miR-15a contributes to these effects. Thus, blockade of the miR-106a∼363 cluster and/or replacement of miR-15a represent possible new strategies for inhibition of Ewing Sarcoma growth.

## Materials and Methods

### Cell Lines and Culture

Ewing Sarcoma cell lines A673, Sk-N-MC, Sk-ES-1 and RD-ES were obtained from ATCC. Ewing Sarcoma cell lines EWS502 and TC71 were obtained from Steve Lessnick at the University of Utah. A673, Sk-N-MC, Sk-ES-1, and EWS502 cells were grown in DMEM supplemented with 10% fetal bovine serum (FBS). TC71 cells were grown in RPMI supplemented with 10% FBS. RD-ES cells were grown in RPMI supplemented with 15% FBS. Low-passage primary human mesenchymal progenitor cells (hMPCs) were obtained from SciCell and Lonza, and cultured in proprietary media.

### MicroRNA Expression Analysis

Total cellular RNA was isolated using the TRIzol reagent, per manufacturer instructions. For each group, RNA was harvested in biological triplicates from plates at similar confluence (50–70%). cDNA was synthesized from 1 µg total cellular RNA using the Qiagen miScript II Reverse Transcriptase kit (Qiagen, Cat #218061), and quantification of microRNA expression levels was performed using the Qiagen SYBRgreen qRT-PCR system (Qiagen, Cat #218075). The relative degree of miR expression between cell lines for a single miR was calculated using the equation: (2^ΔCt^) where ΔCt = (Ct_x miR_ – Ct_U6_). In order to correct for primer efficiency and compare the absolute level of different miRs within a cell line, the best fit linear equation generated by the amplicon standard curve (1.20×10^7^ to 7.71×10^2^ copies/µl) was used to determine the number of copies of the individual miRs and U6 in 333 ng of cDNA as previously describedYoung et al. [57].

### In vitro Cell Growth Assays

For clonogenic assays, EWS cells were plated at a density of 500 cells/well in 6-well plates. After 10–14 days, the cells were washed with PBS and then stained with 0.1% crystal violet in 10% methanol. Colonies were quantified using NIS-Elements System Software. For anchorage-independent colony formation assays, 50,000 cells/well in 6-well plates were grown in 0.35% agar (Difco Agar Noble (BD 214230) and growth medium containing 20% FBS. Colonies were stained with Nitroblue Tetrazolium Chloride, as previously described [4] and quantified using the NIS-Elements System Software. Statistical significance between the control group(s) and the indicated treatments was determined based on an unpaired student’s t-test.

### Transient miR Inhibition and Overexpression Experiments

For transient miR blockade experiments, cells were transfected with a negative control or specific miR hairpin inhibitors (20 nM; Dharmacon), or, alternatively, negative control or specific miR LNAs (100 nM; Exiqon), using Lipofectamine 2000 reagent in both cases. Cell growth experiments and dual renilla/luciferase assays were plated one day after transfection. For rescue experiments, cells expressing s-Neg or s-α-miR-106a∼363 were transfected with 50 nM miR-15a-targeting or negative control LNA (Exiqon) using Lipofectamine 2000 reagent. For transient miR overexpression experiments, cells were transfected with 25 nM miR-15a mimic or negative control (Qiagen) using Lipofectamine 2000 reagent.

### Dual Luciferase Reporter Assay

Cells were seeded in 24-well plates one day after they were transfected with the given LNAs. Three days after seeding, cells were transfected with 200 ng of the psiCHECK2 plasmid (Promega) containing a miR-binding site in the 3′ UTR of renilla for the targeted miR, or a non-targeting binding site. The following day, extract luciferase activity was analyzed per the Promega Dual-Luciferase Reporter Assay protocol.

### Stable miR Sponge Experiments

CMV-d2eGFP sponge plasmids for CXCR4 and miR-18 were obtained from the Sharp lab at MIT. [37] The CXCR4 control is a non-targeting miR sponge based on a sequence from CXCR4 that no miRNAs are predicted to target, as described [37] and used in a numer of studies [58]; in our manuscript, we refer to this control sponge as s-Neg. MiR targeting sequences with a nucleotide bulge were designed as described [37] and obtained from Integrated DNA Technologies for the miR-17∼92a, miR-106b∼25, and/or miR-106a∼363 clusters and the miR-19, miR-20, and miR-25 families. Complementary oligos were annealed and subcloned into CMV-d2eGFP, using standard molecular methods and sequence verified. The bulged miR-binding sites were then subcloned into the pGreen-lentiviral expression vector (System Biosciences), using standard molecular methods and sequence verified. Infectious virus was prepared as previously described. [4] EWS cells were infected with similar titers of virus and selected with Puromycin (2 µg/ml for A673, and 0.5 µg/ml for Sk-ES-1, TC71, and RD-ES). Following 3–7 days of selection, cells were used for experiments and RNA was harvested to check sponge expression.

### Gene Expression Profiling

Biological triplicates of Sk-ES-1, RD-ES, and TC71 cells stably transduced with either s-CXCR4 or s-α-miR-106a∼363 were harvested at ∼70–80% confluence. Total RNA was harvested using the Qiagen RNeasy Mini Kit according to the manufacturer’s protocols. RNA concentration was determined spectrophotometrically. The quality and integrity of RNA were verified using the Agilent 2100 Bioanalyzer. For microarray analysis, 250 ng of total RNA was processed using the Whole Transcript Expression kit (Ambion) and Whole Transcript Terminal Labeling kit (Affymetrix). Samples were hybridized to Human Gene 1.1 ST array strips (Affymetrix) and washed, stained, and imaged using the Gene Atlas Personal Microarray System (Affymetrix). Resulting CEL files were RMA normalized and Log2 transformed using Partek Genomics Suite, and differentially expressed genes were identified using Significance Analysis of Microarrays (SAM) version 4.0 in Excel (www-stat.stanford.edu/∼tibs/SAM) with a false discovery rate of 25%. [59] Normalized data were also analyzed using Gene Set Enrichment Analysis Software (http://www.broadinstitute.org/gsea/doc/GSEAUserGuideFrame.html), available as a stand-alone Java application. The expression profiling data discussed in this publication have been deposited in NCBI’s Gene Expression Omnibus and are accessible through GEO Series accession number GSE45205 (http://www.ncbi.nlm.nih.gov/geo/query/acc.cgi?acc=GSE45205).

## Supporting Information

Figure S1 **Comparison of miR inhibition using a Hairpin Inhibitor or LNA targeting miR-19b.** Dual Renilla/Luciferase assay with miR-19 target in the 3′ UTR of renilla. Sk-ES-1 cells were transfected with 20 nM of a negative control HI, 20 nM of a miR-19b targeting HI, or 50 nM of a miR-19b targeting LNA. Results represent the mean and standard deviation of two experiments performed in triplicate.
(TIF)

Figure S2 **Effects of individual miR blockade on Ewing Sarcoma clonogenic growth.** (A–C) Clonogenic assay in Sk-ES-1 (A), TC71 (B), or Sk-N-Mc (C) cells transfected with 20 nM of a negative control hairpin inhibitor or a hairpin inhibitor (HI) targeting miR-25, miR-93, or miR-25 and miR-93. (D) Dual Renilla/Luciferase assay performed in Sk-ES-1 cells transfected with 20 nM HI-miR-25 and/or HI-miR-93, and the psiCHECK2 dual luciferase reporter with a corresponding complementary binding site in the 3′ UTR of Renilla. (E) Clonogenic assay in Sk-ES-1 cells transfected with 100 nM of a negative control LNA or an LNA targeting miR-17, miR-19b, or miR-92a. (F) Dual Renilla/Luciferase performed in Sk-ES-1 cells transfected with LNA-miR-17, miR-19b, or miR-92a, and the psiCHECK2 dual luciferase reporter with a corresponding complementary binding site in the 3′UTR of renilla. All values represent the mean and SEM of a minimum of two independent experiments, each performed in triplicate.
(TIF)

Figure S3 **Seed sequence mutation of miR-106a∼363 binding sites abolishes growth inhibitory effects of s-α-miR-106a∼363.** (A) Sponge expression was determined in Sk-ES-1 cells stably transduced with s-Neg, s-α-miR-106a∼363^seed mut.^ or s-α-miR-106a∼363 by qRT-PCR. Results represent the mean and SEM of three independent experiments, each performed in triplicate. (B) Clonogenic assay in Sk-ES-1 cells stably expressing s-CXCR4, s-α-miR-106a∼363^seed mut.^, or s-α-miR-106a∼363. Results represent the average and SEM of three independent experiments, each performed in triplicate. *p<0.05 compared to s-Empty according to an unpaired student’s t-test.
(TIF)

Figure S4 **Global gene expression changes in response to s-α-miR-106a∼363 expression in Sk-ES-1 cells.** Affymetrix whole transcript array profiling of s-α-miR-106a∼363 and s-Neg expressing Sk-ES-1 cells. Top upregulated genes (identified by SAM analysis with q-value<25%) upon s-α-miR-106a∼363 expression compared to s-Neg expression are shown.
(TIF)

## References

[1] Riggi N, Suva ML, Stamenkovic I (2009) Ewing's sarcoma origin: from duel to duality. Expert Rev Anticancer Ther 9: 1025–1030. 19671021 10.1586/era.09.81

[2] Arvand A, Denny CT (2001) Biology of EWS/ETS fusions in Ewing's family tumors. Oncogene 20: 5747–5754. 11607824 10.1038/sj.onc.1204598

[3] Toomey EC, Schiffman JD, Lessnick SL (2010) Recent advances in the molecular pathogenesis of Ewing's sarcoma. Oncogene 29: 4504–4516. 20543858 10.1038/onc.2010.205 PMC3555143

[4] McKinsey EL, Parrish JK, Irwin AE, Niemeyer BF, Kern HB, et al (2011) A novel oncogenic mechanism in Ewing sarcoma involving IGF pathway targeting by EWS/Fli1-regulated microRNAs. Oncogene 30: 4910–4920. 21643012 10.1038/onc.2011.197 PMC4696862

[5] Ban J, Jug G, Mestdagh P, Schwentner R, Kauer M, et al (2011) Hsa-mir-145 is the top EWS-FLI1-repressed microRNA involved in a positive feedback loop in Ewing's sarcoma. Oncogene 30: 2173–2180. 21217773 10.1038/onc.2010.581 PMC4959567

[6] Franzetti GA, Laud-Duval K, Bellanger D, Stern MH, Sastre-Garau X, et al.. (2012) MiR-30a-5p connects EWS-FLI1 and CD99, two major therapeutic targets in Ewing tumor. Oncogene. 10.1038/onc.2012.403 22986530

[7] De Vito C, Riggi N, Cornaz S, Suva ML, Baumer K, et al (2012) A TARBP2-dependent miRNA expression profile underlies cancer stem cell properties and provides candidate therapeutic reagents in Ewing sarcoma. Cancer Cell 21: 807–821. 22698405 10.1016/j.ccr.2012.04.023

[8] De Vito C, Riggi N, Suva ML, Janiszewska M, Horlbeck J, et al (2011) Let-7a is a direct EWS-FLI-1 target implicated in Ewing's sarcoma development. PLoS One 6: e23592. 21853155 10.1371/journal.pone.0023592 PMC3154507

[9] Esiashvili N, Goodman M, Marcus RB Jr (2008) Changes in incidence and survival of Ewing sarcoma patients over the past 3 decades: Surveillance Epidemiology and End Results data. J Pediatr Hematol Oncol 30: 425–430. 18525458 10.1097/MPH.0b013e31816e22f3

[10] Ludwig JA (2008) Ewing sarcoma: historical perspectives, current state-of-the-art, and opportunities for targeted therapy in the future. Curr Opin Oncol 20: 412–418. 18525337 10.1097/CCO.0b013e328303ba1d

[11] Subbiah V, Anderson P (2011) Targeted Therapy of Ewing's Sarcoma. Sarcoma 2011: 686985. 21052545 10.1155/2011/686985 PMC2968715

[12] Huang HJ, Angelo LS, Rodon J, Sun M, Kuenkele KP, et al (2011) R1507, an anti-insulin-like growth factor-1 receptor (IGF-1R) antibody, and EWS/FLI-1 siRNA in Ewing's sarcoma: convergence at the IGF/IGFR/Akt axis. PLoS One 6: e26060. 22022506 10.1371/journal.pone.0026060 PMC3191161

[13] Borinstein SC, Barkauskas DA, Krailo M, Scher D, Scher L, et al (2011) Investigation of the insulin-like growth factor-1 signaling pathway in localized Ewing sarcoma: a report from the Children's Oncology Group. Cancer 117: 4966–4976. 21480204 10.1002/cncr.26112 PMC4008340

[14] Frieden M, Orum H (2008) Locked nucleic acid holds promise in the treatment of cancer. Curr Pharm Des 14: 1138–1142. 18473860 10.2174/138161208784246234

[15] Rayner KJ, Esau CC, Hussain FN, McDaniel AL, Marshall SM, et al (2011) Inhibition of miR-33a/b in non-human primates raises plasma HDL and lowers VLDL triglycerides. Nature 478: 404–407. 22012398 10.1038/nature10486 PMC3235584

[16] Huynh C, Segura MF, Gaziel-Sovran A, Menendez S, Darvishian F, et al (2011) Efficient in vivo microRNA targeting of liver metastasis. Oncogene 30: 1481–1488. 21102518 10.1038/onc.2010.523

[17] Fabian MR, Sonenberg N, Filipowicz W (2010) Regulation of mRNA translation and stability by microRNAs. Annu Rev Biochem 79: 351–379. 20533884 10.1146/annurev-biochem-060308-103103

[18] Sotiropoulou G, Pampalakis G, Lianidou E, Mourelatos Z (2009) Emerging roles of microRNAs as molecular switches in the integrated circuit of the cancer cell. RNA 15: 1443–1461. 19561119 10.1261/rna.1534709 PMC2714746

[19] Olive V, Jiang I, He L (2010) mir-17–92, a cluster of miRNAs in the midst of the cancer network. Int J Biochem Cell Biol 42: 1348–1354. 20227518 10.1016/j.biocel.2010.03.004 PMC3681296

[20] Matsubara H, Takeuchi T, Nishikawa E, Yanagisawa K, Hayashita Y, et al (2007) Apoptosis induction by antisense oligonucleotides against miR-17-5p and miR-20a in lung cancers overexpressing miR-17–92. Oncogene 26: 6099–6105. 17384677 10.1038/sj.onc.1210425

[21] Landais S, Landry S, Legault P, Rassart E (2007) Oncogenic potential of the miR-106–363 cluster and its implication in human T-cell leukemia. Cancer Res 67: 5699–5707. 17575136 10.1158/0008-5472.CAN-06-4478

[22] Wang Z, Liu M, Zhu H, Zhang W, He S, et al.. (2012) miR-106a Is frequently upregulated in gastric cancer and inhibits the extrinsic apoptotic pathway by targeting FAS. Mol Carcinog. 10.1002/mc.21899 22431000

[23] Thapa DR, Li X, Jamieson BD, Martinez-Maza O (2011) Overexpression of microRNAs from the miR-17–92 paralog clusters in AIDS-related non-Hodgkin's lymphomas. PLoS One 6: e20781. 21698185 10.1371/journal.pone.0020781 PMC3116840

[24] Reichek JL, Duan F, Smith LM, Gustafson DM, O'Connor RS, et al (2011) Genomic and clinical analysis of amplification of the 13q31 chromosomal region in alveolar rhabdomyosarcoma: a report from the Children's Oncology Group. Clin Cancer Res 17: 1463–1473. 21220470 10.1158/1078-0432.CCR-10-0091 PMC3060277

[25] Olive V, Bennett MJ, Walker JC, Ma C, Jiang I, et al (2009) miR-19 is a key oncogenic component of mir-17–92. Genes Dev 23: 2839–2849. 20008935 10.1101/gad.1861409 PMC2800084

[26] Petrocca F, Vecchione A, Croce CM (2008) Emerging role of miR-106b-25/miR-17–92 clusters in the control of transforming growth factor beta signaling. Cancer Res 68: 8191–8194. 18922889 10.1158/0008-5472.CAN-08-1768

[27] Poliseno L, Salmena L, Riccardi L, Fornari A, Song MS, et al (2010) Identification of the miR-106b∼25 microRNA cluster as a proto-oncogenic PTEN-targeting intron that cooperates with its host gene MCM7 in transformation. Sci Signal 3: ra29. 20388916 10.1126/scisignal.2000594 PMC2982149

[28] Nakatani F, Ferracin M, Manara MC, Ventura S, Del Monaco V, et al (2012) miR-34a predicts survival of Ewing's sarcoma patients and directly influences cell chemo-sensitivity and malignancy. J Pathol 226: 796–805. 21960059 10.1002/path.3007

[29] Dews M, Fox JL, Hultine S, Sundaram P, Wang W, et al (2010) The myc-miR-17∼92 axis blunts TGF{beta} signaling and production of multiple TGF{beta}-dependent antiangiogenic factors. Cancer Res 70: 8233–8246. 20940405 10.1158/0008-5472.CAN-10-2412 PMC3007123

[30] Fu X, Tian J, Zhang L, Chen Y, Hao Q (2012) Involvement of microRNA-93, a new regulator of PTEN/Akt signaling pathway, in regulation of chemotherapeutic drug cisplatin chemosensitivity in ovarian cancer cells. FEBS Lett 586: 1279–1286. 22465665 10.1016/j.febslet.2012.03.006

[31] Jiang Y, Wu Y, Greenlee AR, Wu J, Han Z, et al (2011) miR-106a-mediated malignant transformation of cells induced by anti-benzo[a]pyrene-trans-7,8-diol-9,10-epoxide. Toxicol Sci 119: 50–60. 20889678 10.1093/toxsci/kfq306

[32] Kan T, Sato F, Ito T, Matsumura N, David S, et al (2009) The miR-106b-25 polycistron, activated by genomic amplification, functions as an oncogene by suppressing p21 and Bim. Gastroenterology 136: 1689–1700. 19422085 10.1053/j.gastro.2009.02.002 PMC2887605

[33] Manni I, Artuso S, Careccia S, Rizzo MG, Baserga R, et al (2009) The microRNA miR-92 increases proliferation of myeloid cells and by targeting p63 modulates the abundance of its isoforms. FASEB J 23: 3957–3966. 19608627 10.1096/fj.09-131847

[34] Nittner D, Lambertz I, Clermont F, Mestdagh P, Kohler C, et al (2012) Synthetic lethality between Rb, p53 and Dicer or miR-17–92 in retinal progenitors suppresses retinoblastoma formation. Nat Cell Biol 14: 958–965. 22864477 10.1038/ncb2556

[35] O'Donnell KA, Wentzel EA, Zeller KI, Dang CV, Mendell JT (2005) c-Myc-regulated microRNAs modulate E2F1 expression. Nature 435: 839–843. 15944709 10.1038/nature03677

[36] Mu P, Han YC, Betel D, Yao E, Squatrito M, et al (2009) Genetic dissection of the miR-17∼92 cluster of microRNAs in Myc-induced B-cell lymphomas. Genes Dev 23: 2806–2811. 20008931 10.1101/gad.1872909 PMC2800095

[37] Ebert MS, Neilson JR, Sharp PA (2007) MicroRNA sponges: competitive inhibitors of small RNAs in mammalian cells. Nat Methods 4: 721–726. 17694064 10.1038/nmeth1079 PMC3857099

[38] Dai L, Wang W, Zhang S, Jiang Q, Wang R, et al (2012) Vector-based miR-15a/16-1 plasmid inhibits colon cancer growth in vivo. Cell Biol Int 36: 765–770. 22574716 10.1042/CBI20110404

[39] Diniz MG, Gomes CC, de Castro WH, Guimaraes AL, De Paula AM, et al (2012) miR-15a/16-1 influences BCL2 expression in keratocystic odontogenic tumors. Cell Oncol (Dordr) 35: 285–291. 22684875 10.1007/s13402-012-0087-3 PMC12994990

[40] Klein U, Lia M, Crespo M, Siegel R, Shen Q, et al (2010) The DLEU2/miR-15a/16-1 cluster controls B cell proliferation and its deletion leads to chronic lymphocytic leukemia. Cancer Cell 17: 28–40. 20060366 10.1016/j.ccr.2009.11.019

[41] Ofir M, Hacohen D, Ginsberg D (2011) MiR-15 and miR-16 are direct transcriptional targets of E2F1 that limit E2F-induced proliferation by targeting cyclin E. Mol Cancer Res. 9: 440–447. 10.1158/1541-7786.MCR-10-0344 21454377

[42] Pouliot LM, Chen YC, Bai J, Guha R, Martin SE, et al (2012) Cisplatin Sensitivity Mediated by WEE1 and CHK1 Is Mediated by miR-155 and the miR-15 Family. Cancer Res 72: 5945–5955. 22942255 10.1158/0008-5472.CAN-12-1400 PMC3500396

[43] Lindow M, Kauppinen S (2012) Discovering the first microRNA-targeted drug. J Cell Biol 199: 407–412. 23109665 10.1083/jcb.201208082 PMC3483128

[44] Ventura A, Young AG, Winslow MM, Lintault L, Meissner A, et al (2008) Targeted deletion reveals essential and overlapping functions of the miR-17 through 92 family of miRNA clusters. Cell 132: 875–886. 18329372 10.1016/j.cell.2008.02.019 PMC2323338

[45] Gonzalez I, Vicent S, de Alava E, Lecanda F (2007) EWS/FLI-1 oncoprotein subtypes impose different requirements for transformation and metastatic activity in a murine model. J Mol Med (Berl) 85: 1015–1029. 17453169 10.1007/s00109-007-0202-5

[46] de Alava E, Kawai A, Healey JH, Fligman I, Meyers PA, et al (1998) EWS-FLI1 fusion transcript structure is an independent determinant of prognosis in Ewing's sarcoma. J Clin Oncol 16: 1248–1255. 9552022 10.1200/JCO.1998.16.4.1248

[47] Bandres E, Malumbres R, Escalada A, Cubedo E, Gonzalez I, et al (2005) Gene expression profile of ewing sarcoma cell lines differing in their EWS-FLI1 fusion type. J Pediatr Hematol Oncol 27: 537–542. 16217257 10.1097/01.mph.0000184576.38835.e2

[48] van Doorninck JA, Ji L, Schaub B, Shimada H, Wing MR, et al (2010) Current treatment protocols have eliminated the prognostic advantage of type 1 fusions in Ewing sarcoma: a report from the Children's Oncology Group. J Clin Oncol 28: 1989–1994. 20308669 10.1200/JCO.2009.24.5845 PMC2860404

[49] Liu Q, Fu H, Sun F, Zhang H, Tie Y, et al (2008) miR-16 family induces cell cycle arrest by regulating multiple cell cycle genes. Nucleic Acids Res 36: 5391–5404. 18701644 10.1093/nar/gkn522 PMC2532718

[50] Bonci D, Coppola V, Musumeci M, Addario A, Giuffrida R, et al (2008) The miR-15a-miR-16-1 cluster controls prostate cancer by targeting multiple oncogenic activities. Nat Med 14: 1271–1277. 18931683 10.1038/nm.1880

[51] Mackintosh C, Garcia-Dominguez DJ, Ordonez JL, Ginel-Picardo A, Smith PG, et al.. (2012) WEE1 accumulation and deregulation of S-phase proteins mediate MLN4924 potent inhibitory effect on Ewing sarcoma cells. Oncogene. 10.1038/onc.2012.153 22641220

[52] Bueno MJ, Gomez de Cedron M, Laresgoiti U, Fernandez-Piqueras J, Zubiaga AM, et al (2010) Multiple E2F-induced microRNAs prevent replicative stress in response to mitogenic signaling. Mol Cell Biol 30: 2983–2995. 20404092 10.1128/MCB.01372-09 PMC2876680

[53] Petrocca F, Visone R, Onelli MR, Shah MH, Nicoloso MS, et al (2008) E2F1-regulated microRNAs impair TGFbeta-dependent cell-cycle arrest and apoptosis in gastric cancer. Cancer Cell 13: 272–286. 18328430 10.1016/j.ccr.2008.02.013

[54] Sampath D, Liu C, Vasan K, Sulda M, Puduvalli VK, et al (2012) Histone deacetylases mediate the silencing of miR-15a, miR-16, and miR-29b in chronic lymphocytic leukemia. Blood 119: 1162–1172. 22096249 10.1182/blood-2011-05-351510 PMC3277352

[55] Pickering BF, Yu D, Van Dyke MW (2011) Nucleolin protein interacts with microprocessor complex to affect biogenesis of microRNAs 15a and 16. J Biol Chem 286: 44095–44103. 22049078 10.1074/jbc.M111.265439 PMC3243533

[56] Tang R, Li L, Zhu D, Hou D, Cao T, et al (2012) Mouse miRNA-709 directly regulates miRNA-15a/16-1 biogenesis at the posttranscriptional level in the nucleus: evidence for a microRNA hierarchy system. Cell Res 22: 504–515. 21862971 10.1038/cr.2011.137 PMC3292299

[57] Young CD, Lewis AS, Rudolph MC, Ruehle MD, Jackman MR, et al (2011) Modulation of glucose transporter 1 (GLUT1) expression levels alters mouse mammary tumor cell growth in vitro and in vivo. PLoS One 6: e23205. 21826239 10.1371/journal.pone.0023205 PMC3149640

[58] Ebert MS, Sharp PA (2010) MicroRNA sponges: progress and possibilities. RNA 16: 2043–2050. 20855538 10.1261/rna.2414110 PMC2957044

[59] Tusher VG, Tibshirani R, Chu G (2001) Significance analysis of microarrays applied to the ionizing radiation response. Proc Natl Acad Sci U S A 98: 5116–5121. 11309499 10.1073/pnas.091062498 PMC33173

