# Supplementary figures and images for: Growth-Promoting Role of the miR-106a∼363 Cluster in Ewing Sarcoma
Source: PLoS One. 2013 Apr 26;8(4):e63032. doi: 10.1371/journal.pone.0063032 (PMC3637464; doi:10.1371/journal.pone.0063032)

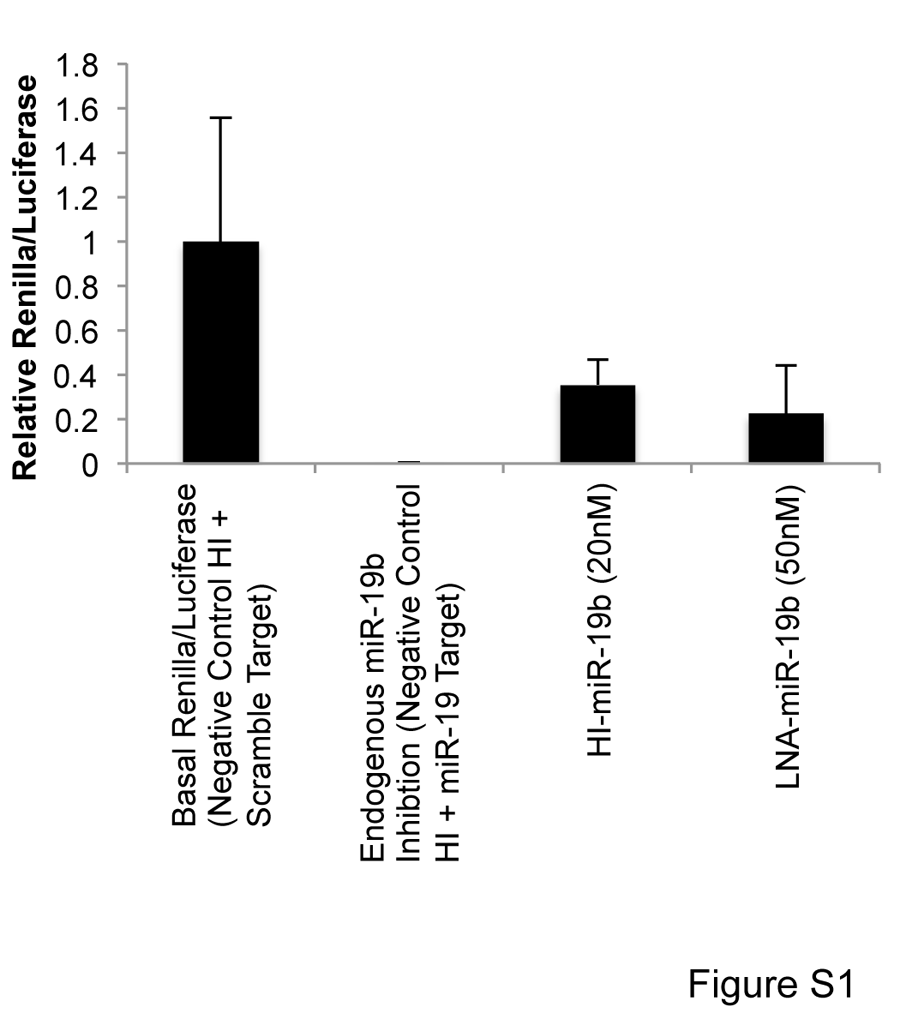

Supplement: Figure S1 — Comparison of miR inhibition using a Hairpin Inhibitor or LNA targeting miR-19b. Dual Renilla/Luciferase assay with miR-19 target in the 3′ UTR of renilla. Sk-ES-1 cells were transfected with 20 nM of a negative control HI, 20 nM of a miR-19b targeting HI, or 50 nM of a miR-19b targeting LNA. Results represent the mean and standard deviation of two experiments performed in triplicate. (TIF) [file pone.0063032.s001.tif]

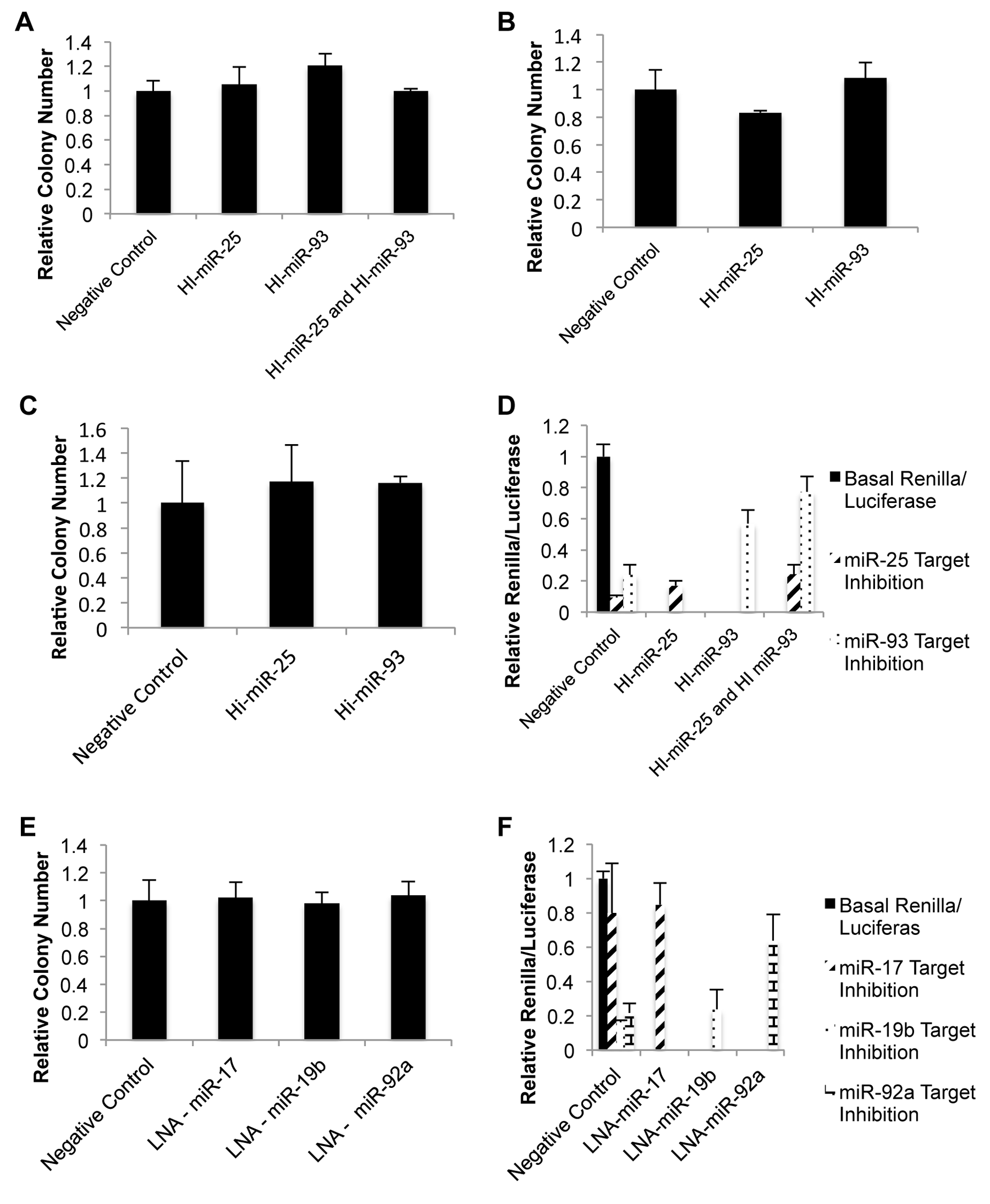

Supplement: Figure S2 — Effects of individual miR blockade on Ewing Sarcoma clonogenic growth. (A–C) Clonogenic assay in Sk-ES-1 (A), TC71 (B), or Sk-N-Mc (C) cells transfected with 20 nM of a negative control hairpin inhibitor or a hairpin inhibitor (HI) targeting miR-25, miR-93, or miR-25 and miR-93. (D) Dual Renilla/Luciferase assay performed in Sk-ES-1 cells transfected with 20 nM HI-miR-25 and/or HI-miR-93, and the psiCHECK2 dual luciferase reporter with a corresponding complementary binding site in the 3′ UTR of Renilla. (E) Clonogenic assay in Sk-ES-1 cells transfected with 100 nM of a negative control LNA or an LNA targeting miR-17, miR-19b, or miR-92a. (F) Dual Renilla/Luciferase performed in Sk-ES-1 cells transfected with LNA-miR-17, miR-19b, or miR-92a, and the psiCHECK2 dual luciferase reporter with a corresponding complementary binding site in the 3′UTR of renilla. All values represent the mean and SEM of a minimum of two independent experiments, each performed in triplicate. (TIF) [file pone.0063032.s002.tif]

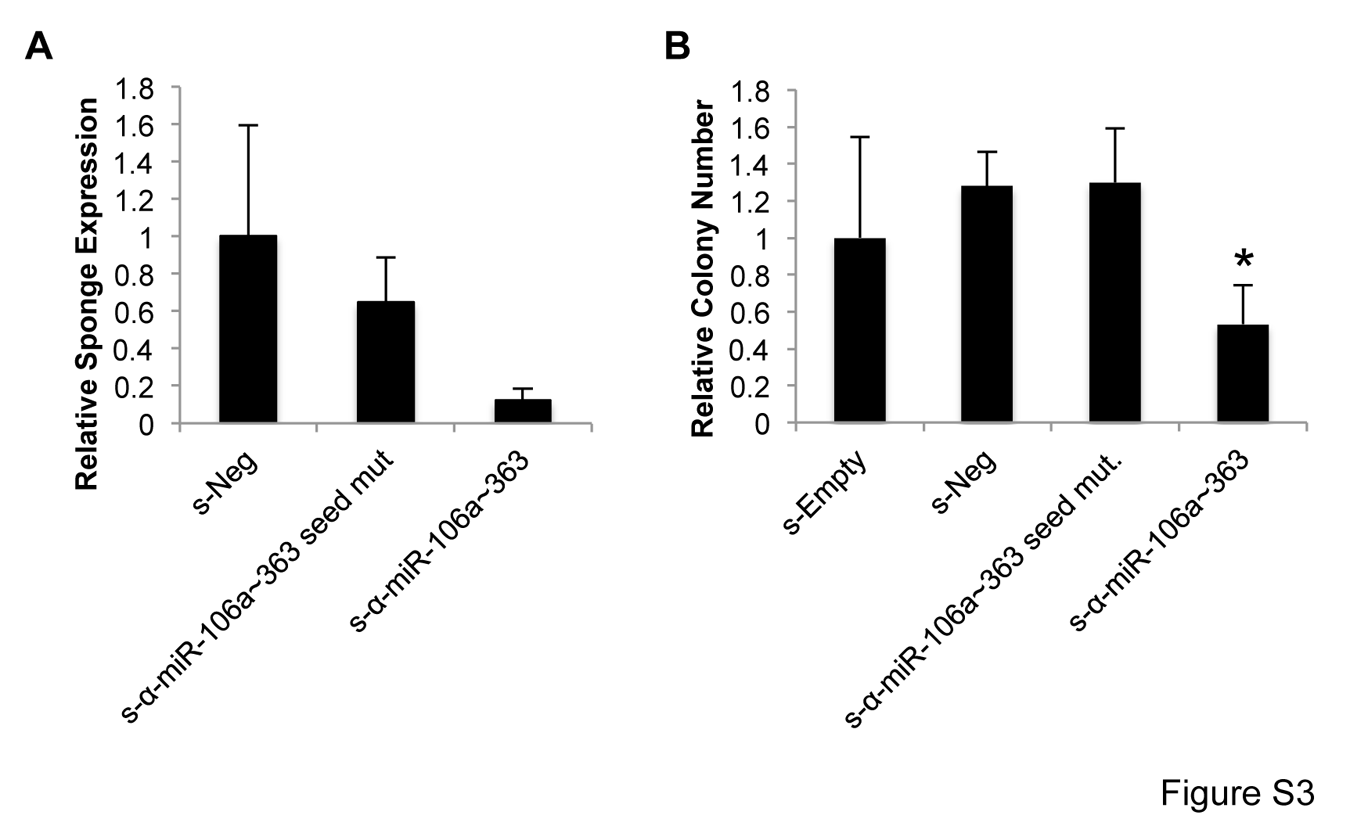

Supplement: Figure S3 — Seed sequence mutation of miR-106a∼363 binding sites abolishes growth inhibitory effects of s-α-miR-106a∼363. (A) Sponge expression was determined in Sk-ES-1 cells stably transduced with s-Neg, s-α-miR-106a∼363seed mut. or s-α-miR-106a∼363 by qRT-PCR. Results represent the mean and SEM of three independent experiments, each performed in triplicate. (B) Clonogenic assay in Sk-ES-1 cells stably expressing s-CXCR4, s-α-miR-106a∼363seed mut., or s-α-miR-106a∼363. Results represent the average and SEM of three independent experiments, each performed in triplicate. *p<0.05 compared to s-Empty according to an unpaired student’s t-test. (TIF) [file pone.0063032.s003.tif]

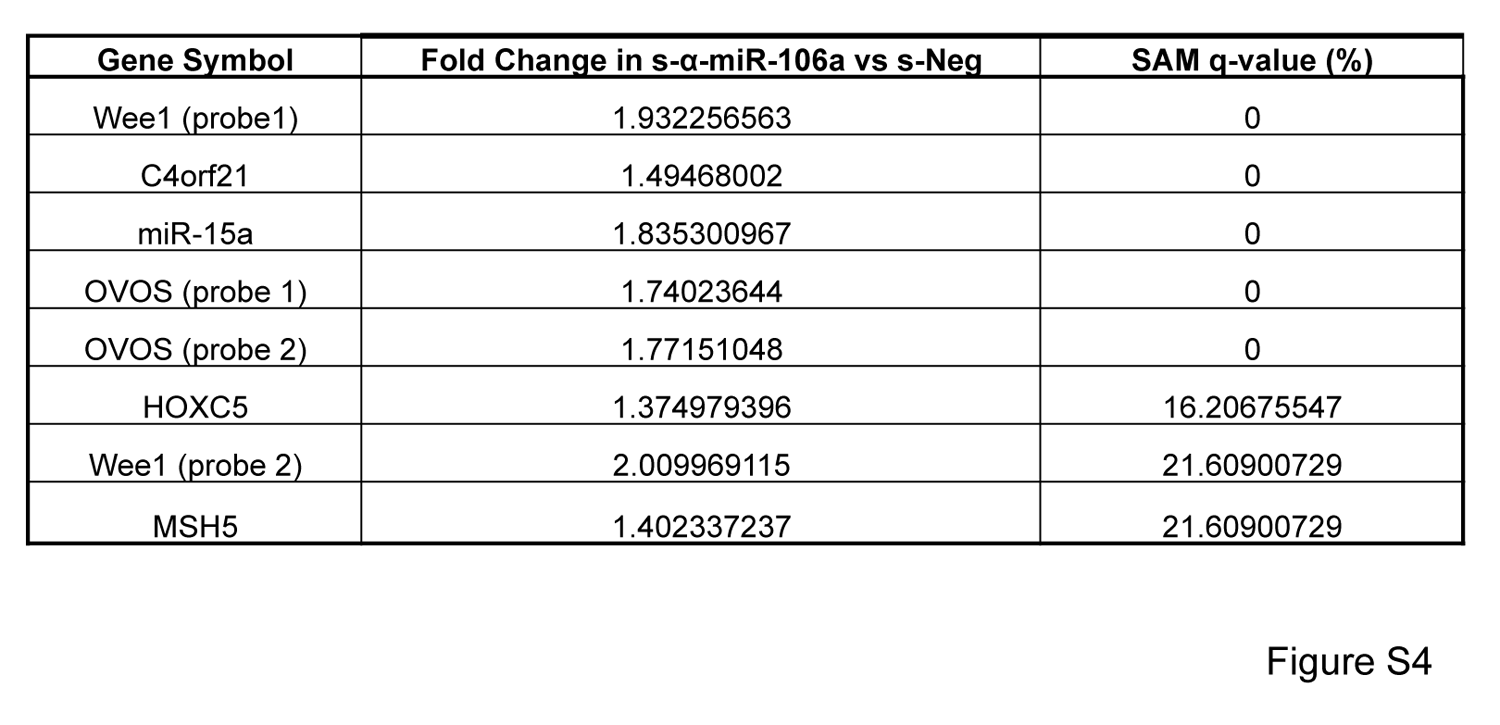

Supplement: Figure S4 — Global gene expression changes in response to s-α-miR-106a∼363 expression in Sk-ES-1 cells. Affymetrix whole transcript array profiling of s-α-miR-106a∼363 and s-Neg expressing Sk-ES-1 cells. Top upregulated genes (identified by SAM analysis with q-value<25%) upon s-α-miR-106a∼363 expression compared to s-Neg expression are shown. (TIF) [file pone.0063032.s004.tif]
